# Analysis of tobacco exposures and high-risk HPV infection in American women: National Health and Nutrition Examination Survey

**DOI:** 10.1007/s11356-023-30175-7

**Published:** 2023-10-04

**Authors:** Liangzi Jiang, Suting Ma, Ge Zhang, Lingling Jiang, Li Yan

**Affiliations:** 1 Department of Obstetrics and Gynecology, The First Affiliated Hospital of Shandong First Medical University & Shandong Provincial Qianfoshan Hospital, Key Laboratory of Laparoscopic Technology, the First Affiliated Hospital of Shandong First Medical University, Jinan, 250000 China; 2 Department of Pediatrics, Linyi People’s Hospital, Linyi, 276000 China; 3 Department of Gastroenterology, Shandong Medical College (Linyi Geriatric Hospital), Linyi, 276000 China

**Keywords:** Tobacco exposure, High-risk human papillomavirus, HR-HPV, NHANES

## Abstract

Nicotine is a known toxin, but its relationship with cervicovaginal high-risk human papillomavirus (HR-HPV) infection is uncertain. This study aimed to investigate whether tobacco exposure is associated with elevated cervicovaginal HR-HPV infection in US women, and if the strength of this association varies with the degree of exposure. Cross-sectional data from the 2011–2016 National Health and Nutrition Examination Survey (NHANES), which included a nationally representative sample of US women, were used for the study. Out of 12436 women aged 18–59 who participated in the interview, 3833 were ultimately enrolled in this study. Weighted logistic regression was used to estimate the link between tobacco exposure and cervicovaginal HR-HPV infection. The mean age of participants was 38.6 (SD 12.1) years, and non-Hispanic White individuals accounted for 37.3% of the sample. Those with any tobacco exposure tended to be younger (mean age 37.7 [SD 12.4] years vs 40.3 [11.2] years), non-Hispanic Black (27.8% vs. 15.1%), lower educated (41.8% vs. 29.4%), and have lower family income (39.9% vs. 23.5%). After adjustment, the odds of having HR-HPV infection were 1.32 (95% CI, 1.09–1.59) for those exposed to tobacco, remaining significant in multiple sensitivity analyses and across subgroups. This study, based on a nationally representative sample from the United States, suggests that tobacco exposure is a risk factor for elevated HR-HPV infection in women, highlighting the need for further research into reducing this modifiable risk factor.

**Supplementary Information:**

The online version contains supplementary material available at 10.1007/s11356-023-30175-7.

## Introduction

As one of the most widespread sexually transmitted diseases, human papillomavirus (HPV) infection poses a significant public health challenge for women across the globe. Persistent infection with high-risk oncogenic HPV (HR-HPV) may give rise to severe cancers like oropharyngeal, penile, vulvovaginal, anal, and, notably, cervical cancer (Shiraz et al. 2022). According to CDC estimates, HPV is responsible for nearly 200,000 diagnoses of cervical precancer and 11,100 cases of cervical cancer annually, resulting in 4,000 deaths from cervical cancer every year in the United States (https://www.cdc.gov/hpv/parents/cancer.html, accessed on April 7, 2023). Developing countries may face an even more challenging situation due to their low socio-economic status and poor health conditions. Therefore, it is critical to explore the underlying factors associated with HR-HPV to prevent or treat it.

Cigarette smoking is a pervasive habit worldwide, and tobacco exposure has been linked to the development of brain, respiratory, cardiovascular, infectious, and malignant diseases (Warren and Cummings 2013; Warren et al. 2014). Despite the extensive body of scientific literature indicating that tobacco use impedes the clearance of HPV, leading to persistent HPV infection (Ciccarese et al. 2021; Kum-Nji et al. 2019; Sadate-Ngatchou et al. 2016; Tarney et al. 2018), a limited number of studies have reported that smoking is not associated with HPV infection (Collins et al. 2010; Kelsey et al. 2015). However, it is important to note that these previous studies predominantly relied on self-reported smoking data or solely measured serum cotinine concentrations as indicators of tobacco exposure. These measurement approaches introduce potential selection bias and may yield unstable findings.

The purpose of this study was to determine the association between tobacco exposure, defined by self-reported smoking status combined with serum cotinine, and HR-HPV infection among a representative sample of adult US women.

## Materials and methods

### Data sources and study population

The National Health and Nutrition Examination Survey (NHANES) is a cross-sectional study conducted every two years to assess the health and nutritional status of non-institutionalized individuals in the United States. Administered by the Centers for Disease Control and Prevention, NHANES is a nationally representative, stratified multistage probability survey. The survey involves a mobile examination center (MEC) that conducts home visits, screenings, and laboratory tests to collect detailed demographic and health data (Zipf et al. 2013). Since 1999, the NHANES project has received approval from the National Center for Health Statistics Institutional Review Board and Ethics Review Board, and all participants must provide written informed consent before participation. Secondary analysis is permitted without further Institutional Review Board permission. All data are available through the NHANES website, and sample weights are used to account for participant selection probability and non-response adjustments. The survey's structure and weighting formula have been previously discussed (Parsons et al. 2014), and this report adheres to the STROBE (Strengthening the Reporting of Observational studies in Epidemiology) reporting guidelines for cross-sectional studies.

For our investigation, we utilized data from three consecutive cycles (2011–2016) of the National Health and Nutrition Examination Survey. Since public records for genital HPV infection are only available for this age group, our analysis included individuals who were 18 years or older but younger than 59 years at the time of participation and completed the required tests. Those who tested positive for pregnancy, reported kidney disease, or had a serum creatinine level greater than 2 mg/dl were excluded from the study due to their possible impact on nicotine metabolism (Benowitz et al. 2009).

### Definition of tobacco exposure

Tobacco exposure was determined by identifying participants who actively smoked, had at least one smoker in their household, or had a serum cotinine level exceeding 0.05 ng/mL. Active smoking was defined as a positive response to the question, ' During the past 5 days, including today, did you smoke cigarettes, pipes, cigars, little cigars or cigarillos, water pipes, hookahs, or e-cigarettes?' or by having a serum cotinine level greater than 10 ng/mL. Participants exposed to tobacco but not actively smoking were classified as passive smoking. A 0.05 ng/mL lower cutoff level, initially established based on the assay limit of detection, is still widely accepted as standard in recent literature examining tobacco exposure.

The Division of Laboratory Sciences, National facility for Environmental Health, Centers for Disease Control and Prevention in Atlanta, Georgia examined all samples. Participants provided serum samples at the mobile examination center, and a method utilizing isotope-dilution high-performance liquid chromatography and tandem atmospheric pressure chemical ionization mass spectrometry was used to determine serum cotinine levels (Bernert et al. 1997). The lower limit of detection for this assay is 0.015 ng/mL, and it exhibits high accuracy with mean values within 9% of theoretical values at all levels, except for the lowest limit of quantification, where they are within 14%. Furthermore, the assay has high precision, with a within-day coefficient of variation less than 5% at every concentration, except for the lower limit of quantification, where it is less than 16%.

### Outcomes

Participants were instructed to self-collect cervicovaginal samples at the mobile examination center, which were analyzed for extracted HPV DNA using the Linear Array HPV Genotyping Assay (Hariri et al. 2011). The NHANES provided results for the detection of HPV DNA for 37 different HR-HPV types. If a participant had any of the 14 HR-HPV genotypes (16, 18, 31, 33, 35, 39, 45, 51, 52, 56, 58, 59, 66, or 68), they were considered to have an HR-HPV infection. Participants who tested positive for β-globin controls but had none of the above HPV types were considered negative for HR-HPV infection. Samples that tested negative for HR-HPV and had negative β-globin controls were unsuitable for analysis, while participants who did not provide or had inadequate cervicovaginal samples were excluded from analysis.

### Covariates

According to the literature (Brouwer et al. 2019; Tarney et al. 2016, 2018), various possible factors were assessed, including age, race/ethnicity, education, marital status, family income, body mass index (BMI), alcohol consumption, health insurance, vaginal deliveries, age of first sexual activity, number of lifetime sexual partners, and HPV vaccine status. Race/ethnicity categories included Mexican American, Other Hispanic, non-Hispanic white, non-Hispanic black, and Other Race, which encompassed multi-racial individuals. Education was classified as high school graduate or less, some college, or college graduate or above. Marital status was coded as married or living with a partner, widowed, divorced, or separated, or never married. Family income was grouped into low (PIR ≤ 1.3), medium (PIR > 1.3 to 3.5), and high (PIR > 3.5) categories according to the poverty income ratio (PIR) from a US government report (https://www.ars.usda.gov/northeast-area/beltsville-md-bhnrc/beltsville-human-nutrition-research-center/food-surveys-research-group/docs/wweia-data-tables/, accessed on March 1, 2023). BMI was calculated based on participants' weight in kilograms and height in meters. Alcohol consumption was indicated by drinking at least 12 alcoholic beverages per year, including liquor, beer, wine, wine coolers, and other alcoholic beverages. Participants with neither private nor public insurance were coded as having no insurance. Vaginal deliveries referred to the number of deliveries during the interview. Age of first sexual activity was defined as the age at which participants had their first oral, anal, or vaginal intercourse. Lifetime sexual partners referred to the number of men with whom participants had vaginal, anal, or oral intercourse throughout their lives. HPV vaccine status was determined by whether participants had received either of the two available HPV vaccines, Cervarix and Gardasil.

### Statistical analysis

This is a secondary analysis of freely available datasets. Mean and standard deviation (SD) values were used to define continuous variables that were normally distributed, while median values and interquartile ranges (IQR) were used for non-normally distributed variables. Categorical variables were expressed as proportions (%). For group comparisons, either the t-test (for normally distributed data), Mann–Whitney test (for skewed distributions), or χ2 test (for categorical variables) were used as appropriate. To assess the association between tobacco exposure and HR-HPV infection, multivariable logistic regression was used to calculate the odds ratios (OR) and 95% confidence intervals (95% CIs). Age, race/ethnicity, education, marital status, family income, BMI, alcohol consumption, insurance status, age of first sexual activity, lifetime sexual partners, HPV vaccine status, and vaginal deliveries were all included in the final model.

Furthermore, sensitivity analyses were conducted using cotinine as a continuous variable, with the limit of detection of cotinine as a threshold for exposure, and smoking status classified as none (cotinine < 0.05 ng/ml and no self-reported exposure), passive (cotinine 0.05–10 ng/ml or self-reported household smoke exposure), or active (cotinine > 10 ng/ml or self-reported current smoker). The effect of the natural logarithm of cotinine on HR-HPV infection odds ratio, after adjusting for confounders, was examined.

Moreover, following the adjustment of variables in Model 3, a restricted cubic spline (RCS) regression with four knots at the 5th, 35th, 65th, and 95th percentiles of the natural logarithm of cotinine was performed to investigate the relationship between cotinine and HR-HPV infection in a dose–response fashion.

Subgroup analyses were conducted to explore potential modifications of the relationship between tobacco exposure and HR-HPV infection. The following variables were investigated: age (< 25 vs ≥ 25 years), education level (high school graduate or some college or above), marital status (married or living with a partner vs. widowed, divorced, separated, or never married), family income (low vs. medium or high), BMI (< 25 vs. ≥ 25 kg/m2), alcohol consumption, health insurance, first age (< 17 vs. ≥ 17 years), lifetime sexual partners (< 5 vs. ≥ 5), and HPV vaccine status.

No a priori statistical power estimations were conducted because the sample size was determined solely based on the available information. Statistical analysis was performed using the R-4.0.2 software package (http://www.R-project.org, The R Foundation) and Free Statistics software versions 1.71, with appropriate sampling weights to account for the complex survey design. Two-sided tests were used for all P values, and results were considered statistically significant if P < 0.05.

## Results

### Basic characteristics

**Fig. 1:**
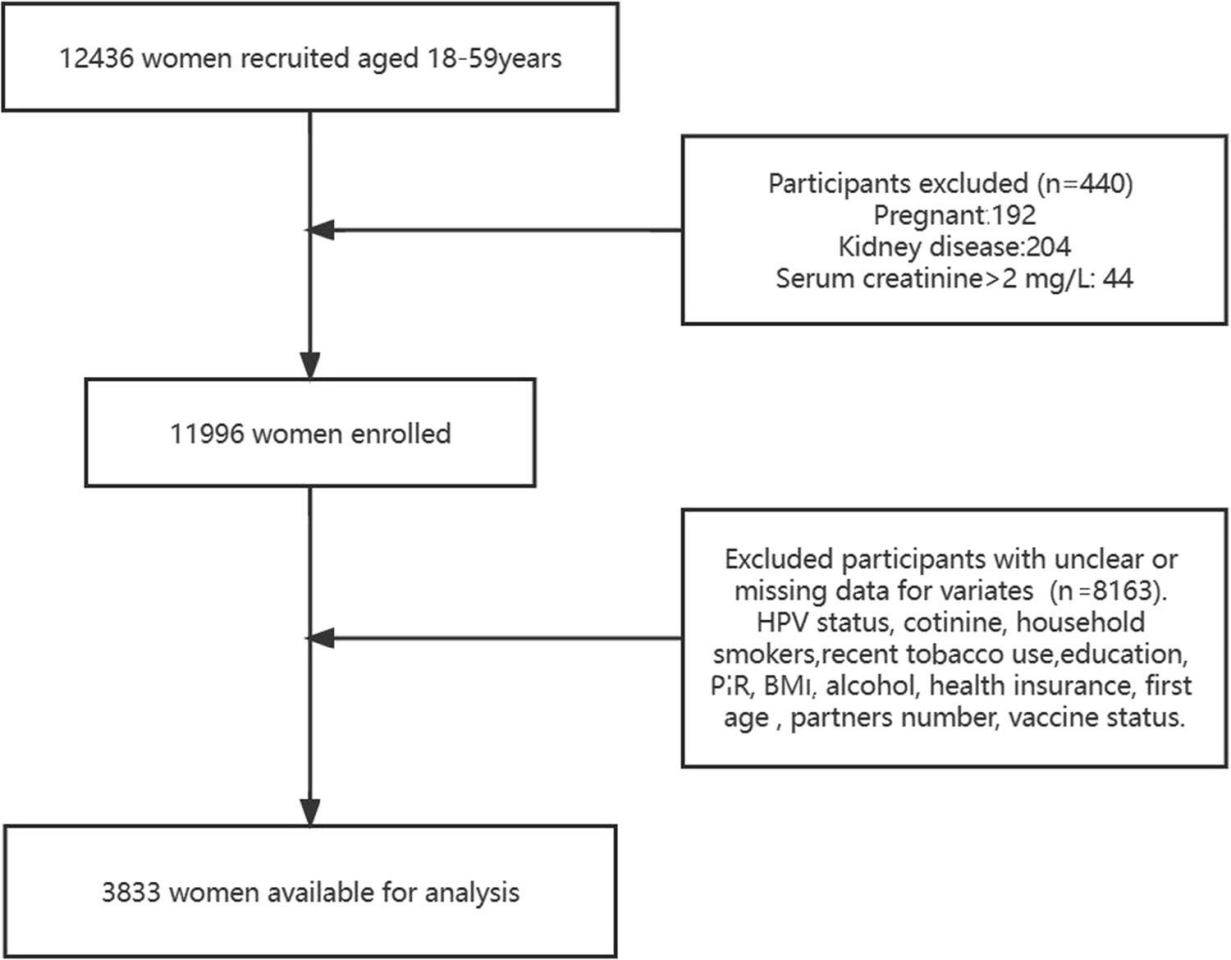
eps The study’s flow diagram. Figure 1 was created at this website: https://www.processon.com

A total of 12,436 individuals aged 18 to 59 participated in the interview. Among them, we excluded 440 women, consisting of 192 pregnant women, 204 with kidney disease, and 44 with serum creatine levels > 2 mg/L. Additionally, those missing data on HR-HPV status, tobacco exposure, serum cotinine, education level, family income, BMI, alcohol consumption, health insurance, first age, lifetime sexual partners, or HPV vaccine status were further excluded (*n* = 8163). Eventually, 3833 participants from NHANES conducted between 2011 and 2016 were included in the analysis. Further details regarding the selection process are presented in Fig. 1.

Among the 3833 participants included in this study, the mean age was 38.6 (12.1) years, and 37.3% were non-Hispanic White. Table 1 presents the baseline characteristics of the selected individuals based on their tobacco exposure status. Of the total sample, 2508 (65.4%) reported tobacco exposure. Participants with any form of tobacco exposure tended to be younger (mean [SD] age, 37.7 [12.4] vs. 40.3 [11.2] years), more likely to be non-Hispanic Black (27.8% vs. 15.1%), have lower education level (41.8% vs. 29.4%) and family income (39.9% vs. 23.5%), consume alcohol, have no health insurance, report greater number of sexual partners, and exhibit higher serum cotinine levels.

**Table 1:** Population characteristics by categories of tobacco exposure

**Characteristic**	**Total**	**Tobacco exposure**		**P-value**
		**None**	**Any**	
	***n* = 3833**	***n* = 1325**	***n* = 2508**	
Age(year),Mean ± SD	38.6 ± 12.1	40.3 ± 11.2	37.7 ± 12.4	< 0.001
Race/ethnicity, n (%)				< 0.001
Mexican American	544 (14.2)	283 (21.4)	261 (10.4)	
Other Hispanic	426 (11.1)	172 (13)	254 (10.1)	
Non-Hispanic White	1430 (37.3)	462 (34.9)	968 (38.6)	
Non-Hispanic Black	896 (23.4)	200 (15.1)	696 (27.8)	
Other Race—Including Multi-Racial	537 (14.0)	208 (15.7)	329 (13.1)	
Education, n (%)				< 0.001
High school graduate or less	1438 (37.5)	389 (29.4)	1049 (41.8)	
Some College	1292 (33.7)	399 (30.1)	893 (35.6)	
College Graduate or above	1103 (28.8)	537 (40.5)	566 (22.6)	
Marital status, n (%)				< 0.001
Married or living with a partner	2093 (54.6)	873 (65.9)	1220 (48.6)	
Widowed, divorced, separated	673 (17.6)	200 (15.1)	473 (18.9)	
Never married	1067 (27.8)	252 (19)	815 (32.5)	
Family income, n (%)				< 0.001
Low	1312 (34.2)	311 (23.5)	1001 (39.9)	
Medium	1326 (34.6)	457 (34.5)	869 (34.6)	
High	1195 (31.2)	557 (42)	638 (25.4)	
BMI, Mean ± SD	29.7 ± 8.0	29.4 ± 7.8	29.8 ± 8.1	0.071
HR-HPV status, n (%)				< 0.001
Yes	2975 (77.6)	1118 (84.4)	1857 (74)	
No	858 (22.4)	207 (15.6)	651 (26)	
Alcohol, n (%)				< 0.001
Yes	2618 (68.3)	831 (62.7)	1787 (71.3)	
No	1215 (31.7)	494 (37.3)	721 (28.7)	
Health insurance, n (%)				< 0.001
Yes	2966 (77.4)	1090 (82.3)	1876 (74.8)	
No	867 (22.6)	235 (17.7)	632 (25.2)	
Vaginal deliveries, n (%)				0.003
Less than 1	1309 (34.2)	430 (32.5)	879 (35)	
Greater than 2	1566 (40.9)	590 (44.5)	976 (38.9)	
Missing	958 (25.0)	305 (23)	653 (26)	
HPV vaccine status, n (%)				0.001
Yes	552 (14.4)	157 (11.8)	395 (15.7)	
No	3281 (85.6)	1168 (88.2)	2113 (84.3)	
First age(year), Median (IQR)	17.0 (15.0, 19.0)	18.0 (16.0, 20.0)	16.0 (15.0, 18.0)	< 0.001
Lifetime sexual partners, Median (IQR)	5.0 (2.0, 9.0)	4.0 (2.0, 7.0)	5.0 (3.0, 10.0)	< 0.001
Serum cotinine(ng/mL), Median (IQR)	0.034 (0.011, 2.100)	0.011 (0.011, 0.021)	0.194 (0.026, 102.500)	< 0.001

### Associations between tobacco exposure and HR-HPV infection

Table 2 displays the results of the multivariate analysis, indicating a significant association between HR-HPV infection and tobacco exposure. Compared to those without tobacco exposure, the crude odds ratio (OR) for individuals with any tobacco exposure and HR-HPV infection was 1.89 (95% CI, 1.59–2.25), suggesting that tobacco exposure is a significant risk factor for HR-HPV infection. As we adjusted for more covariates in different models, the ratio was attenuated but continued to be statistically significant (OR, 1.44 [95%CI, 1.20–1.74] in model 1, OR, 1.37 [95%CI, 1.14–1.66] in model 2, and OR, 1.32 [95%CI, 1.09–1.59] in model 3).

**Table 2:** Association between tobacco exposure and HR-HPV infection in multiple regression and sensitivity analyses

Variables	OR (95% CI)							
	**Crude**	**P-value**	**Model 1**	**P-value**	**Model 2**	**P-value**	**Model 3**	**P-value**
No tobacco exposure	1(Ref)		1(Ref)		1(Ref)		1(Ref)	
Any tobacco exposure	1.89 (1.59 ~ 2.25)	< 0.001	1.44 (1.20 ~ 1.74)	< 0.001	1.37 (1.14 ~ 1.66)	0.001	1.32 (1.09 ~ 1.59)	0.004
**Sensitivity analyses**								
Lower detection limit of cotinine (0.015 ng/ml)	1.99 (1.66 ~ 2.39)	< 0.001	1.52 (1.25 ~ 1.85)	< 0.001	1.45 (1.19 ~ 1.77)	< 0.001	1.39 (1.14 ~ 1.7)	0.001
Natural log of cotinine (ng/ml)	1.11 (1.08 ~ 1.13)	< 0.001	1.09 (1.06 ~ 1.11)	< 0.001	1.08 (1.05 ~ 1.1)	< 0.001	1.07 (1.04 ~ 1.09)	< 0.001
Passive tobacco exposure	1.51 (1.25 ~ 1.82)	< 0.001	1.2 (0.98 ~ 1.47)	0.07	1.18 (0.97 ~ 1.45)	0.1	1.17 (0.95 ~ 1.43)	0.134
Active smoking	2.65 (2.17 ~ 3.25)	< 0.001	2.04 (1.63 ~ 2.55)	< 0.001	1.83 (1.46 ~ 2.3)	< 0.001	1.7 (1.35 ~ 2.15)	< 0.001
P value for trend		< 0.001		< 0.001		< 0.001		< 0.001

Abbreviation: HPV, human papillomavirus; OR, odds ratio; CI, confidence interval; Ref: reference

Model 1 was adjusted for sociodemographic variables (age, race/ethnicity, education, marital status, family income)

Model 2 was adjusted for sociodemographic (age, race/ethnicity, education, marital status, family income), BMI, alcohol, health insurance

Model 3 was adjusted for sociodemographic (age, race/ethnicity, education, marital status, family income), BMI, alcohol, health insurance, first age, lifetime sexual partners, HPV vaccine status, vaginal deliveries

### Stratified analyses based on additional variables

**Fig. 2:**
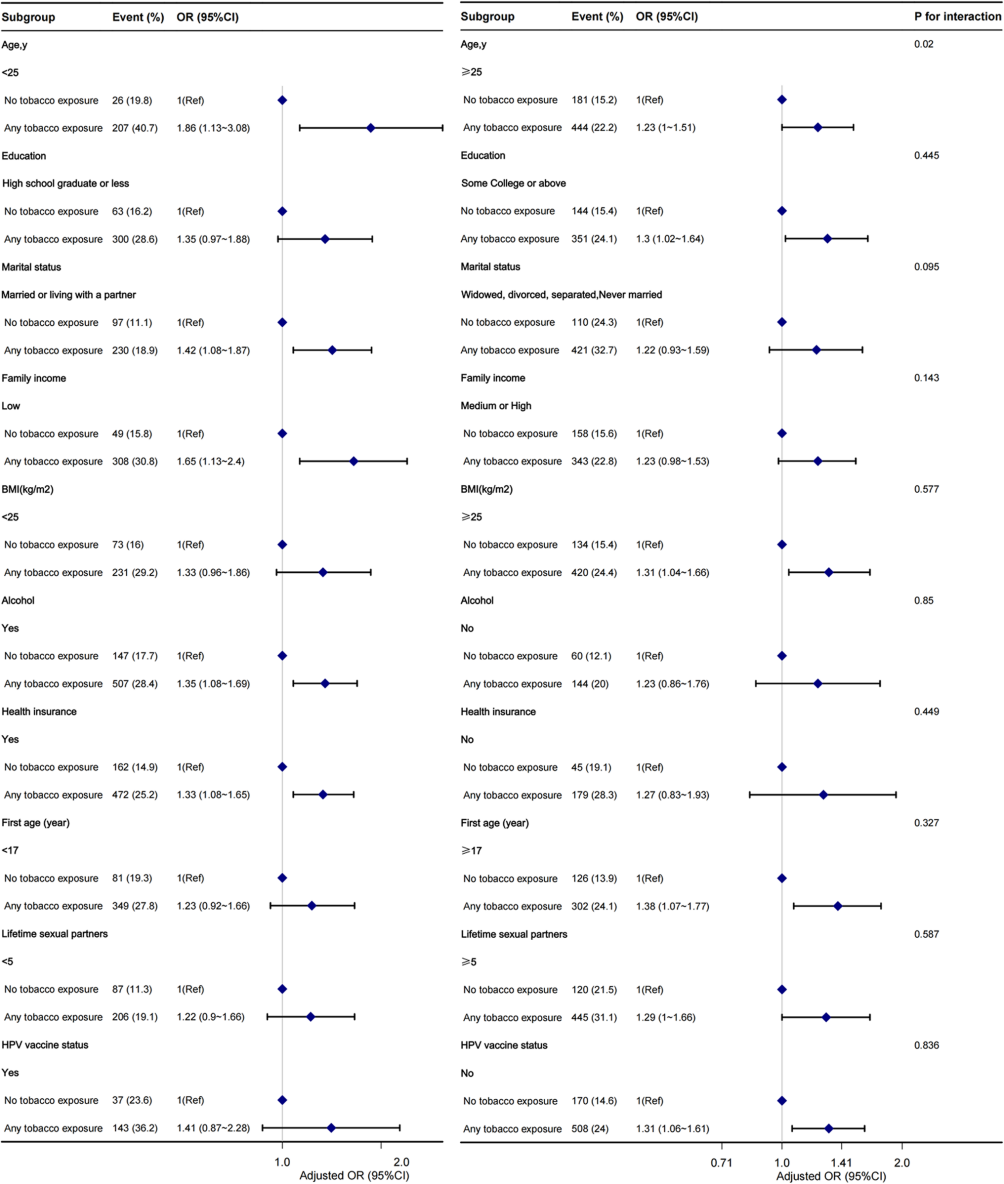
eps The relationship between tobacco exposure and HR-HPV infection according to basic features. Except for the stratification component itself, each stratification factor was adjusted for all other variables (age, race/ethnicity, education, marital status, family income, BMI, alcohol, health insurance, first age, Lifetime sexual partners, HPV vaccine status, vaginal deliveries). Figure 2 and Fig. 3 were created by the statistical software packages R (http://www.R-project.org, The R Foundation)

Figure 2 shows the results of subgroup analyses examining the potential effect modifications on the association between tobacco exposure and HR-HPV infection. There was a significant relationship between tobacco exposure and HR-HPV infection among participants aged less than 25 years (OR, 1.86 [95% CI, 1.13–3.08]), those with higher education (OR, 1.3 [95% CI, 1.02–1.64]), those married or living with a partner (OR, 1.42 [95% CI, 1.08–1.87]), those with low family income (OR, 1.65 [95% CI, 1.13–2.4]), those with BMI ≥ 25 kg/m2 (OR, 1.31 [95% CI, 1.04–1.66]), those who drink alcohol (OR, 1.35 [95% CI, 1.08–1.69]), those with health insurance (OR, 1.33 [95% CI, 1.08–1.65]), those with first age ≥ 17 (OR, 1.38 [95% CI, 1.07–1.77]), and those without HPV vaccine status (OR, 1.31 [95% CI,1.06–1.61]). No significant interactions were found in any subgroups, except those stratified by age. However, a p-value of less than 0.05 for the age interaction may not be statistically significant when adjusting for multiple tests.

### Sensitivity analysis

**Fig. 3:**
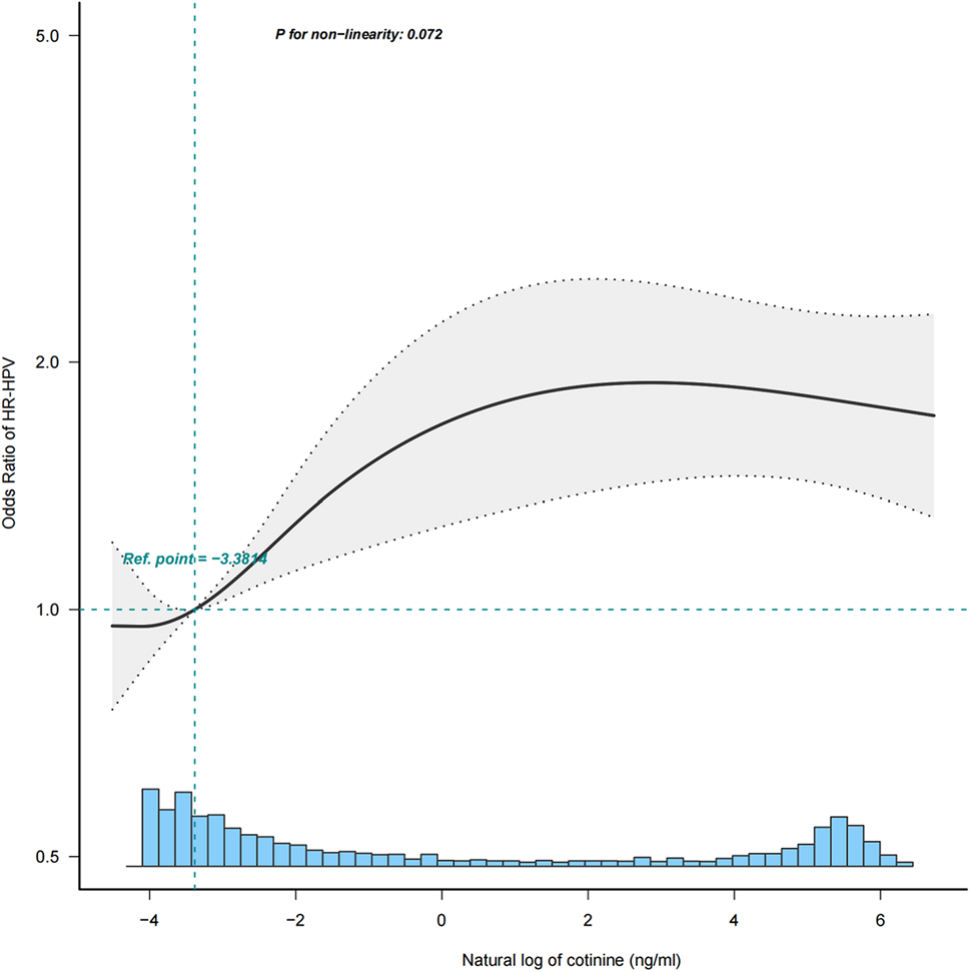
eps Association between natural logarithm of cotinine and HR-HPV infection odds ratio. Covariates: sociodemographic (age, race/ethnicity, education, marital status, family income), BMI, alcohol, health insurance, first age, lifetime sexual partners, HPV vaccine status, vaginal deliveries

After categorizing study subjects based on the lower detection limit of cotinine (0.015 ng/ml), 68.7% were identified as tobacco exposed. Results from Table 2 showed that individuals with tobacco exposure had a crude odds ratio (OR) of 1.99 (95% CI, 1.66–2.39) for HR-HPV infection compared to those without tobacco exposure. Similar outcomes were observed in different adjusted models. Using the natural logarithmically transformed cotinine as a continuous measure of degree of tobacco exposure, a dose–response association was found between degree of tobacco exposure and HR-HPV infection. Specifically, for every increase in the logarithm of cotinine level, the odds of having HR-HPV infection increased by 7% (95% CI, 4%–9%) after adjusting all covariates, as shown in Table 2. This trend is also shown in Fig. 3. Active smoking was persistently associated with HR-HPV infection (OR, 1.70 [95% CI, 1.35–2.15]). While statistical significance was not observed for passive exposure (OR, 1.17 [95% CI, 0.95–1.43]), a statistically significant trend was observed between exposure groups (P < 0.001) (Table 2).

## Discussion

In this extensive cross-sectional study of American women, we found a significant link between tobacco exposure and HR-HPV infection that remained significant even after adjusting for various factors, including age, race/ethnicity, education, marital status, PIR, BMI, alcohol use, health insurance, age at first sexual intercourse, number of lifetime sexual partners, HPV vaccination status, and vaginal deliveries. The association was similar across various subgroups of participants. Moreover, comparable associations were observed in sensitivity analyses that utilized different definitions of smoking status.

The current literature on the association between tobacco exposure and HPV infection, especially HR-HPV, has yielded inconsistent results. Smoking has been linked to an increased risk of cervical dysplasia (Matsumoto et al. 2010; Tsai et al. 2007). Although some studies have found correlations between smoking and HPV infection (Sadate-Ngatchou et al. 2016; Vaccarella et al. 2008), others have not (Collins et al. 2010; Kelsey et al. 2015). It is noteworthy that these studies had non-standard definitions of smoking and did not utilize nationally representative data. In contrast, our study employed biomarkers in combination with self-reported smoking to accurately determine tobacco exposure. Moreover, we restricted our focus to HR-HPV types, which are linked to cervical dysplasia or cancer, thus enhancing the clinical relevance of our findings.

The relationship between tobacco exposure and HR-HPV infection remains unclear. Our findings are consistent with several other studies that have reported a link between smoking and genital HPV infection. Based on the NHANES database, Christopher et al. demonstrated an association between tobacco use and increased odds of high-risk genital HPV infection (Tarney et al. 2018); however, they did not include self-reported smoking as an evaluation parameter for tobacco exposure. In another survey by Philip Kum-Nji et al., both passive and active tobacco smoking status were strongly correlated with any HPV infection in women aged 18 to 26 years, with a significant dose–response relationship (Kum-Nji et al. 2019). Nevertheless, they did not take into account self-reported smoking as an exposure factor nor excluded pregnant participants, those with kidney disease or high creatine levels, which could affect nicotine metabolism. Our study, on the other hand, not only relied on self-reported smoking but also incorporated serum cotinine levels to define tobacco exposure. This approach ensured that our results are more consistent and reliable. Besides, our study is more robust as it included sensitivity analysis and stratification analysis methods, yielding more reliable results. It notes that Fig. 3 and Table 2 both show a strong dose–response relationship between cotinine and HR-HPV infection. However, this relationship is not necessarily causal because of the cross-sectional nature of the data collection procedures. As a metabolite of nicotine, there is no evidence that cotinine is harmful to the body, and there is no literature on its pathogenic mechanisms. So, this finding needs to be further explored. In a prospective investigation by Collins et al., they observed no difference in the risk of HPV infection between smokers and nonsmokers (Collins et al. 2010). The rationale for their conclusion could be that they relied on self-reported smoking status and neglected the passive smokers.

Although the exact mechanism underlying the inverse association between tobacco exposure and HR-HPV infection is still unknown, our results are biologically plausible based on current evidence. Studies have shown that smoking can compromise cellular and humoral immunity, affecting both systemic and local immunity, which may impede HPV clearance and result in prolonged HPV infection (Ciccarese et al. 2021; de Jong et al. 2004). Infections may be triggered through innate and humoral pathways by the numerous toxins present in tobacco smoke (Kum-Nji et al. 2006). Mohan Sopori's review identified nicotine as the principal immunosuppressive component of cigarette smoke, suppressing both innate and adaptive immune responses (Qiu et al. 2017). Nicotine has been demonstrated to inhibit T cell activity and natural killer cells and suppress the production of type-specific HPV antibodies (Kalra et al. 2000; Simen-Kapeu et al. 2008). Cigarette smoke has been shown to exert harmful effects on immune and tissue cells via the regulation of NFκB and MAPK signaling, as well as histone modification (Ahn and Aggarwal 2005; D’Anna et al. 2015; Manzel et al. 2011; Talikka et al. 2012).

Although additional research is necessary to determine if tobacco exposure is causally linked to HR-HPV infection, the findings of this investigation have significant clinical implications. Cervical cancer or dysplasia is predominantly preventable and associated with HR-HPV infection. Targeted measures must be taken to deal with smoking and environmental nicotine in light of the observed correlation between tobacco exposure and HR-HPV infection. Based on the current study, it is evident that active and passive smoke exposure should be prevented to decrease the risk of HR-HPV infection.

Several limitations must be considered when interpreting these findings. First, NHANES only collected HPV data between 2011 and 2016, making it challenging to validate our results utilizing data from other time periods. Second, although we used sensitivity analysis, stratified analyses, and regression models, unmeasured or unidentified covariates may have a residual confounding impact that could not be entirely eliminated. Third, this study was conducted solely on women aged 18–59 years in the United States, and it is uncertain whether our findings can be generalized to men or individuals outside this age range, necessitating further investigation. Finally, due to the inherent limitations of cross-sectional research, causality cannot be concluded, and further confirmation by longitudinal studies is necessary in the future. Aside from the association between HR-HPV infection and tobacco exposure, future studies may also investigate other factors that might affect HR-HPV infection, such as nutrition.

## Conclusions

This study uncovered a link between tobacco exposure and HR-HPV infection in women aged 18–59 years in the United States, which persisted even after factoring in potential confounders. The robustness of sensitivity analyses using alternative exposure definitions and stratified analyses in subgroups supported this relationship. Tobacco exposure is hazardous to numerous bodily systems and may also have detrimental effects on the immune system, resulting in an elevated risk of HR-HPV infection.

### Supplementary Information

Below is the link to the electronic supplementary material.

Supplementary file1 (XLSX 3696 KB)

## Data Availability

All data in the article are accessible (http://www.cdc.gov/nchs/nhanes.htm). To facilitate the reproduction of our results, we provide the list of anonymous patient identifiers for databases in Supplementary data.

## Abbreviations

HPV: Human papillomavirus
HR-HPV: High-risk human papillomavirus
NHANES: National Health and Nutrition Examination Survey
CDC: Centers for Disease Control and Prevention
MEC: Mobile examination center
BMI: Body mass index
PIR: Poverty income ratio
SD: Standard deviation
IQR: Interquartile ranges
OR: Odds ratios
CI: Confidence intervals

## References

[CR1] Ahn KS, Aggarwal BB (2005). Transcription factor NF-kappaB: a sensor for smoke and stress signals. Ann N Y Acad Sci.

[CR2] Benowitz NL, Hukkanen J, Jacob P (2009) Nicotine chemistry, metabolism, kinetics and biomarkers. Handb Exp Pharmacol 29–60. 10.1007/978-3-540-69248-5_2 10.1007/978-3-540-69248-5_2 PMC2953858 19184645

[CR3] Bernert JT, Turner WE, Pirkle JL (1997). Development and validation of sensitive method for determination of serum cotinine in smokers and nonsmokers by liquid chromatography/atmospheric pressure ionization tandem mass spectrometry. Clin Chem.

[CR4] Brouwer AF, Eisenberg MC, Carey TE, Meza R (2019). Multisite HPV infections in the United States (NHANES 2003–2014): An overview and synthesis. Prev Med.

[CR5] Ciccarese G, Herzum A, Pastorino A (2021). Prevalence of genital HPV infection in STI and healthy populations and risk factors for viral persistence. Eur J Clin Microbiol Infect Dis.

[CR6] Collins S, Rollason TP, Young LS, Woodman CBJ (2010). Cigarette smoking is an independent risk factor for cervical intraepithelial neoplasia in young women: a longitudinal study. Eur J Cancer.

[CR7] D’Anna C, Cigna D, Costanzo G (2015). Cigarette smoke alters cell cycle and induces inflammation in lung fibroblasts. Life Sci.

[CR8] de Jong A, van Poelgeest MIE, van der Hulst JM (2004). Human papillomavirus type 16-positive cervical cancer is associated with impaired CD4+ T-cell immunity against early antigens E2 and E6. Cancer Res.

[CR9] Hariri S, Unger ER, Sternberg M (2011). Prevalence of genital human papillomavirus among females in the United States, the National Health And Nutrition Examination Survey, 2003–2006. J Infect Dis.

[CR10] Kalra R, Singh SP, Savage SM (2000). Effects of cigarette smoke on immune response: chronic exposure to cigarette smoke impairs antigen-mediated signaling in T cells and depletes IP3-sensitive Ca(2+) stores. J Pharmacol Exp Ther.

[CR11] Kelsey KT, Nelson HH, Kim S (2015). Human papillomavirus serology and tobacco smoking in a community control group. BMC Infect Dis.

[CR12] Kum-Nji P, Meloy L, Herrod HG (2006). Environmental tobacco smoke exposure: prevalence and mechanisms of causation of infections in children. Pediatrics.

[CR13] Kum-Nji P, Meloy L, Keyser-Marcus L (2019). Tobacco smoke exposure as a risk factor for human papillomavirus infections in women 18–26 years old in the United States. PLoS One.

[CR14] Manzel LJ, Shi L, O’Shaughnessy PT (2011). Inhibition by cigarette smoke of nuclear factor-κB-dependent response to bacteria in the airway. Am J Respir Cell Mol Biol.

[CR15] Matsumoto K, Oki A, Furuta R (2010). Tobacco smoking and regression of low-grade cervical abnormalities. Cancer Sci.

[CR16] Parsons VL, Moriarity C, Jonas K (2014). Design and estimation for the national health interview survey, 2006–2015. Vital Health Stat.

[CR17] Qiu F, Liang C-L, Liu H (2017). Impacts of cigarette smoking on immune responsiveness: Up and down or upside down?. Oncotarget.

[CR18] Sadate-Ngatchou P, Carter JJ, Hawes SE (2016). Determinants of High-Risk Human Papillomavirus Seroprevalence and DNA Prevalence in Mid-Adult Women. Sex Transm Dis.

[CR19] Shiraz A, Egawa N, Pelt DM (2022). Cervical cell lift: A novel triage method for the spatial mapping and grading of precancerous cervical lesions. EBioMedicine.

[CR20] Simen-Kapeu A, Kataja V, Yliskoski M (2008). Smoking impairs human papillomavirus (HPV) type 16 and 18 capsids antibody response following natural HPV infection. Scand J Infect Dis.

[CR21] Talikka M, Sierro N, Ivanov NV (2012). Genomic impact of cigarette smoke, with application to three smoking-related diseases. Crit Rev Toxicol.

[CR22] Tarney CM, Klaric J, Beltran T (2016). Prevalence of Human Papillomavirus in Self-Collected Cervicovaginal Swabs in Young Women in the United States Between 2003 and 2012. Obstet Gynecol.

[CR23] Tarney CM, Beltran TA, Klaric J, Han JJ (2018). Tobacco Use and Prevalence of Human Papillomavirus in Self-Collected Cervicovaginal Swabs Between 2009 and 2014. Obstet Gynecol.

[CR24] Tsai H-T, Tsai Y-M, Yang S-F (2007). Lifetime cigarette smoke and second-hand smoke and cervical intraepithelial neoplasm–a community-based case-control study. Gynecol Oncol.

[CR25] Vaccarella S, Herrero R, Snijders PJF (2008). Smoking and human papillomavirus infection: pooled analysis of the International Agency for Research on Cancer HPV Prevalence Surveys. Int J Epidemiol.

[CR26] Warren GW, Sobus S, Gritz ER (2014). The biological and clinical effects of smoking by patients with cancer and strategies to implement evidence-based tobacco cessation support. Lancet Oncol.

[CR27] Warren GW, Cummings KM (2013) Tobacco and lung cancer: risks, trends, and outcomes in patients with cancer. Am Soc Clin Oncol Educ Book 359–364. 10.14694/EdBook_AM.2013.33.359 10.14694/EdBook_AM.2013.33.359 23714547

[CR28] Zipf G, Chiappa M, Porter KS (2013). National health and nutrition examination survey: plan and operations, 1999–2010. Vital Health Stat.

